# Overlimiting current near a nanochannel a new insight using molecular dynamics simulations

**DOI:** 10.1038/s41598-021-94477-x

**Published:** 2021-07-26

**Authors:** D. Manikandan, Vishal V. R. Nandigana

**Affiliations:** Fluid Systems Laboratory, Department of Mechanical Engineering, Indian Institute of Technology Madras, Chennai, 600036 India

**Keywords:** Mechanical engineering, Nanofluidics, Computational nanotechnology

## Abstract

In this paper, we report for the first time overlimiting current near a nanochannel using all-atom molecular dynamics (MD) simulations. Here, the simulated system consists of a silicon nitride nanochannel integrated with two reservoirs. The reservoirs are filled with ${0.1} \, \hbox {M}$ potassium chloride (KCl) solution. A total of ${\sim } 1.1$ million atoms are simulated with a total simulation time of ${\sim } 1 {\mu s}$ over ${\sim }$ 30000 CPU hours using 128 core processors (Intel(R) E5-2670 2.6 GHz Processor). The origin of overlimiting current is found to be due to an increase in chloride (${Cl^-}$) ion concentration inside the nanochannel leading to an increase in ionic conductivity. Such effects are seen due to charge redistribution and focusing of the electric field near the interface of the nanochannel and source reservoir. Also, from the MD simulations, we observe that the earlier theoretical and experimental postulations of strong convective vortices resulting in overlimiting current are not the true origin for overlimiting current. Our study may open up new theories for the mechanism of overlimiting current near the nanochannel interconnect devices.

## Introduction

Nanochannels are used for many applications such as seawater desalination^1,2^, biomolecule preconcentration^3,4^, nanofluidic diode^5–8^, and DNA sequencing^9,10^. Nanochannels are typically integrated with two reservoirs and a potential difference is applied across the reservoirs. The ion selectivity of the nanochannel is controlled by the surface charge density of the nanochannel and thickness of the electrical double layer (EDL)^11–13^. When the solid wall of the nanochannel comes into contact with electrolyte solution, the surface charge density of the nanochannel induces an electric potential. Ions are redistributed according to the potential and form an electrical double layer (EDL). The thickness of the EDL is given by the Debye length, ${\lambda _d = \sqrt{\epsilon _0\epsilon _rRT/2F^2z^2c_0}}$, where ${\epsilon _0}$ is the permittivity of free space, ${\epsilon _r}$ is the relative permittivity of the medium, *R* is the ideal gas constant, *T* is temperature, *F* is the Faraday’s constant, *z* is the valency of the ions and $c_0$ is the bulk concentration. Typically, the thickness of the EDL can be controlled by varying the bulk concentration of the solution. At low bulk concentrations, the thickness of the EDL will be of the order of few nanometers and if the height of the nanochannel is comparable to EDL thickness, the EDLs overlap and result in predominant transport of counter-ions through the nanochannel^11–16^. In this way, the nanochannel act as an ion exchange membrane.

**Figure 1:**
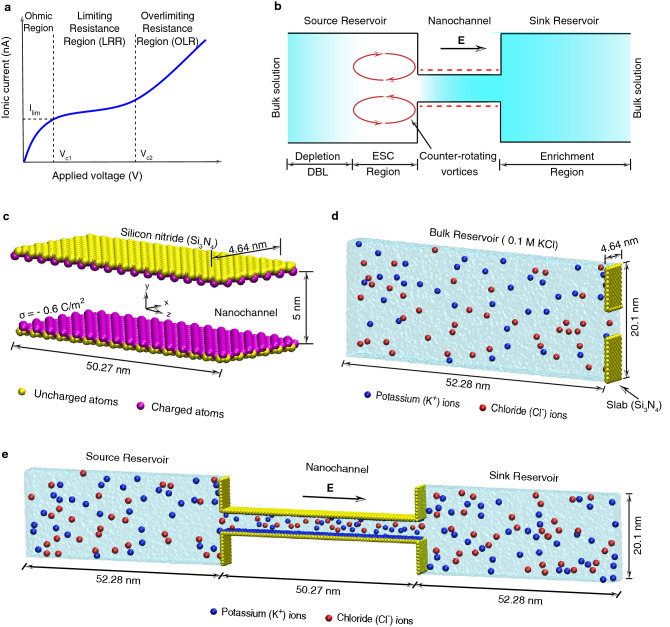
(**a**) The schematic diagram of the typical I-V behaviour of the ion exchange devices. (**b**) shows the sketch of the various regions in the nanochannel integrated system under non-ohmic regions. (**c**) shows a silicon nitride nanochannel of height 5 nm used in all-atom MD simulations. To obtain the desired surface charge ($\sigma ={-0.6} \, {C/m^2}$) of the nanochannel, the additional charges are added to the surface atoms along with its partial charge. (**d**) shows the bulk reservoir filled with 0.1 M potassium chloride (KCl) solution. The silicon nitride slab is used to guide the flow through the nanochannel from the bulk reservoir. (**e**) The final assembled view of the simulation system. Semitransparent surface shows the volume filled with water molecules. For clear visualization, we showed only a few number of ions in the system. Dimensions in the figure show the size of the system after 5 ns of NPT simulation. Drawings in (**c**–**e**) were created using VMD software (www.ks.uiuc.edu/Development/Download/download.cgi?PackageName=VMD and version 1.9.4).

The application of potential difference across the nanochannel creates an imbalance between cation and anion flux leading to the formation of the depletion and enrichment of the electrolyte solution across the interface of the nanochannel resulting in ion concentration polarization (ICP)^17–34^. The ion concentration polarization (ICP) effect leads to nonlinear I-V characteristics resulting in three regions, namely, Ohmic region, limiting resistance region (LRR) and overlimiting resistance region (OLR) (see Fig. 1a). The Ohmic region is linear and agrees with the Ohm’s law. In limiting resistance region (LRR), the formation of the extended space charge (ESC) region at the depletion side of the nanochannel (see Fig. 1b) limits the current and we observe I-V slope lower than the Ohmic region. However, the current increases linearly again when the voltage is increased and this region is called overlimiting resistance region (OLR)^32,33^.

Owing to the advancements in micro/nanofabrication technology, many experimental investigations have been conducted to understand the finite resistance in the limiting resistance region and the transition from the limiting resistance region to the overlimiting resistance region^17–26^. It is observed that the formation of strong convective vortices in the depletion side of the nanochannel decreases the length of the electro-neutral depletion diffusion boundary layer (DBL) (see Fig. 1b) that results in the overlimiting resistance region. But, recent continuum scale numerical investigations by Nandigana and Aluru^32,33^ showed that the strong convective vortices may not be responsible for the overlimiting current. To find out the true mechanism, here, we report all-atom molecular dynamics (MD) simulations^35–39^ to understand ion concentration polarization (ICP) and overlimiting current near a nanochannel for the first time.

## Non linear I–V characteristics

A typical simulation system includes a silicon nitride ($Si_3N_4$) nanochannel (see Fig. 1c) integrated with two reservoirs filled with 0.1M potassium chloride (KCl) solution (see Fig. 1d). The final assembled simulation system and the direction of the applied electric field is shown in Fig. 1e. Based on the direction of the applied electric field, we classify the left and right side reservoirs as the source and sink reservoirs, respectively.

**Figure 2:**
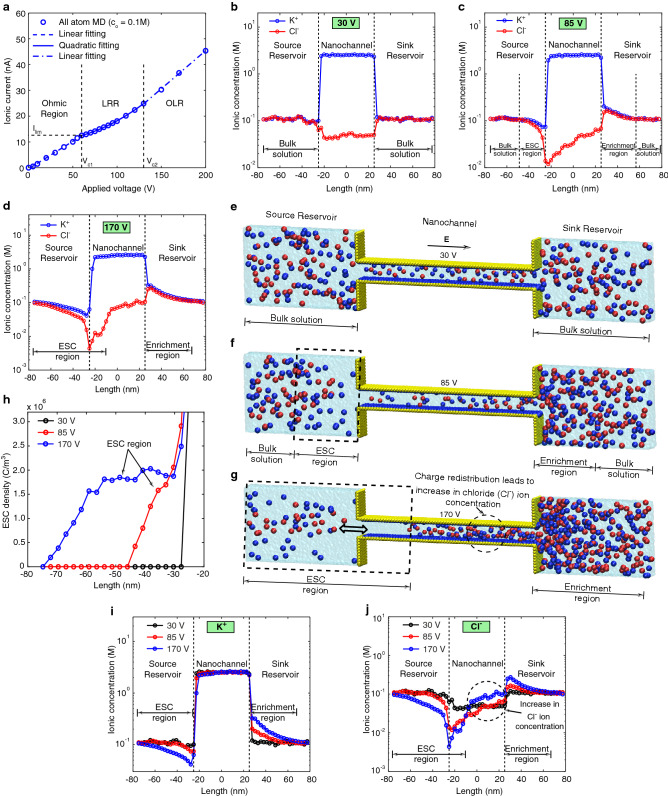
(**a**) reveals the nonlinear I–V characteristics of the system from all-atom MD simulations. It is divided into three regions, similar to experiments, namely, Ohmic region, limiting resistance region (LRR), and overlimiting resistance region (OLR). (**b–d**) shows the concentration distribution of ions along the length of the system for all three regions, corresponding to the applied voltage of 30V, 85V, and 170V respectively and (**e–g**) shows corresponding snapshots from the all-atom MD simulations. For clear visualization we shown only ions inside the system within a range of x = -1.0 to 1.0 nm and z = -59 to z = 59 nm. Also, the arrow shows the direction of propagation of the ESC region. Semitransparent surface indicates the volume occupied by the water molecules. (**h**) shows the variation of the ESC density inside the source reservoir. (**i–j**) shows the comparison of the concentration distribution of potassium ($K^+$) and chloride ($Cl^-$) ions in all three regions, respectively. Drawings in (**e**–**g**) were created using VMD software (www.ks.uiuc.edu/Development/Download/download.cgi?PackageName=VMD and version 1.9.4).

In this work, we considered the length of the simulation set up to be 154.8 nm and each simulation run for 10 ns. Also, we assumed the surface charge density of the nanochannel to be ${-0.6} \, {C/m^2}$, to capture all three regions of the I-V characteristics by overcoming the limitations of the molecular dynamics (MD) simulations in studying the low electric field (${< 10^8} \, V/m$) dynamics^37,40^. Recently, such a high surface charge density is reported inside the nanofluidic devices^41^. The overall system comprises ${\sim } 1.1$ million atoms (see Supplementary Table. 1). The simulation is performed for a total simulation time of ${\sim } 1 \mu s$ over ${\sim }$ 30000 CPU hours using 128 core processors (Intel(R) E5-2670 2.6 GHz Processor). A voltage range of 5 V - 200 V is applied across the ends of the reservoirs. The ionic current is measured inside the nanochannel^9,36,37^ using Eq. 1,

1 $$\begin{aligned} I (t+ \Delta t /2) = \frac{1}{\Delta t L_n} \sum _{i=1}^{N} q_i (z_i(t+\Delta t) - z_i(t)) \end{aligned}$$

where, ${i} = {1,2, \ldots ,N}$, *N* is the number of ions inside the nanochannel, ${L_n}$ is the length of the nanochannel, ${z_i(t)}$ and ${z_i(t+\Delta t)}$ is the position of each ion at *t* and ${t+\Delta t}$ time step respectively, ${\Delta t} = 0.1 \, \hbox {ps}$, and ${q_i}$ is the charge of each ion. Our simulations showed that during the initial period, the current decays with time and after 1-2 ns the current fluctuates around a steady-state value (see Supplementary Fig. 1). We attribute the fluctuations in the current to the thermal motion of ions. The current is averaged in the steady-state region and the result is shown in Fig. 2a.

Figure 2a reveals the nonlinear I-V characteristics showing all three regions, namely, Ohmic region, limiting resistance region (LRR), and overlimiting resistance region (OLR).

### Ohmic region

In the Ohmic region, the current varies linearly with the applied voltage following the Ohm’s law (see Fig. 2a). Here, ions are electro-neutral in both the source and sink reservoirs, but we observe higher potassium (${K^+}$) ion concentration than chloride (${Cl^-}$) ion concentration inside the nanochannel owing to the nanochannel negative surface charge density (see Fig. 2b,e and Supplementary Movie. 1). Also, we observe a thin electrical double layer (EDL) at the interface of the nanochannel and the source and sink reservoirs. In addition, the high negative surface charge density of the nanochannel allows more potassium ions to be adsorbed on the surface. These adsorbed potassium ions overscreen the surface charge density and attract chloride ions near the surface, causing charge inversion^42–44^. However, when we increase the applied voltage ($> \, \hbox {30V}$), the ions that overscreen the surface charge density are driven away, resulting in the dominant transport of potassium ions inside the nanochannel^44,45^. Further, most of the voltage drop occurs across the nanochannel making the nanochannel resistance as the main controlling resistance for the current. Furthermore, we observe that the electric field is high inside the nanochannel compared to both the reservoirs because of high electrostatic interactions between the adsorbed cations and negatively charged wall atoms.

### Limiting resistance region (LRR)

In the limiting resistance region, the current deviates from the Ohm’s law, but the current does not saturate as predicted by the classical diffusion-limited current transport theory but increases slowly with applied voltage. The results agree well with the literature^20–23^. As we increase the voltage above the Ohmic region, the potassium ions continue to enter the nanochannel from the source reservoir as the nanochannel is selective to the potassium ions, whereas the chloride ions are driven away from the interface of the nanochannel and source reservoir. It results in the depletion of ions, and the formation of the extended space charge (ESC) region at the interface of the nanochannel and source reservoir. In the meantime, at the interface of the nanochannel and sink reservoir, the chloride ions are blocked and accumulated due to the ion selectivity of the nanochannel. To maintain electro-neutrality, the potassium ions are also accumulated, leading to the formation of the enrichment region inside the sink reservoir (see Fig. 2c,f and Supplementary Movie. 2). Extended space charge (ESC) region is a non-zero net charge region and is formed because of the ion selectivity of the nanochannel and the normal component of the electric field. Fig. 2h shows the net charge density inside the source reservoir for all three regions. In contrast to the Ohmic region, the formation and propagation of the ESC region inside the source reservoir creates a non-uniformity in the electric field distribution and makes the electric field focused near the interface of the nanochannel and source reservoir. Also, the ESC region increases the resistance of the source reservoir and makes it comparable to the nanochannel. On the other hand, we do not observe any non-uniformity in the electric field distribution inside the sink reservoir owing to the presence of the electroneutral solution (see Fig. 2c and f). Moreover, the ESC region acts as a barrier to the flow of ions into the nanochannel and provides finite resistance to the ionic current resulting in the limiting resistance region.

**Figure 3:**
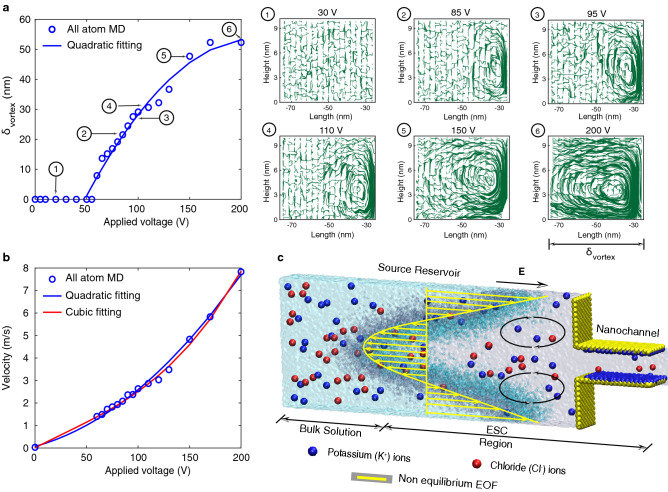
(**a**) indicates the variation of the size of the counter-rotating vortices ($\delta _{vortex}$) with the applied voltage. Further, it shows the fluid flow streamlines for the six different voltages. The counter-rotating vortices are almost symmetry about the centre of the source reservoir. For clear visualization, we show one-half of the vortices. (**b**) shows the variation of the velocity of the water inside the source reservoir with the applied voltage. It follows the quadratic scaling with the applied voltage similar to the earlier theoretical and experimental results. (**c**) illustrates the snapshot of the counter-rotating vortices from all-atom MD simulation, corresponding to the overlimiting resistance region. Drawing in (**c**) was created using VMD software (www.ks.uiuc.edu/Development/Download/download.cgi?PackageName=VMD and version 1.9.4).

The normal component of the electric field on the ESC region leads to the formation of counter-rotating vortices near the interface of the nanochannel and source reservoir (see Fig. 3a). In order to maintain the continuity of the fluid flow from the source reservoir to the nanochannel, some portion of the fluid is redirected back at the centre of the source reservoir forming counter-rotating vortices (see Fig. 3c and Supplementary Movie. 4). Fig. 3a shows the variation of the size of the counter-rotating vortices ($\delta _{vortex}$) with applied voltage and also shows the fluid flow streamlines for six different voltages. Further increase in applied voltage, the vortices continue to propagate towards the source reservoir (see Fig. 3a) and that leads to an increase in velocity (see Fig. 3b).

### Overlimiting resistance region (OLR)

In the overlimiting resistance region, the current again increases linearly like the Ohmic region. We observe the propagation of the ESC region towards the source reservoir and the enrichment region towards the sink reservoir (see Fig. 2d). The propagation of the ESC region towards the source reservoir increases the strength of the normal component of the focused electric field. Further, this focused electric field allows the ESC region to propagate into the nanochannel. The charge redistribution mechanism due to the ESC propagation towards the source reservoir and into the nanochannel changes the chloride ($Cl^-$) ion concentration inside the nanochannel resulting in an increase in ionic conductivity and causes the overlimiting current (see Fig. 2j and Supplementary Movie. 3). Fig. 2g shows the charge redistribution mechanism as a snapshot taken from all-atom MD simulations. The potassium ($K^+$) ion concentration for all three regions is shown in Fig. 2i. We do not observe any significant changes in the potassium ion concentration inside the nanochannel due to the high negative surface charge density.

Similar to the limiting resistance region, we observe counter-rotating vortices inside the source reservoir. The strong convective vortices grow with the applied voltage and propagate and occupy the entire source reservoir (see Fig. 3a and Supplementary Movie. 4). As we continue to increase the voltage further, the size of the vortices ($\delta _{vortex}$) saturate because they reach the end of the source reservoir. The results are consistent with the experimental observations in the literature^22,23^. Also, the velocity of the counter-rotating vortices follows a quadratic scaling with the electric field (see Fig. 3b) as given by Rubinstein’s non-equilibrium electro-osmotic flow (EOF) formulation (${u_{EOF} = - \frac{1}{8} E^2 \frac{\partial ^2 c}{\partial y \partial z} / \frac{\partial c}{\partial z}}$)^17,46,47^ .

To test if convective vortices are the reason for the overlimiting current, we performed a hypothetical MD simulation with KCl molecules (powder) and not KCl solution (KCl + water). For the hypothetical MD simulation, the dielectric constant was set to be 79 (See Methods section). Supplementary Fig. 2a shows the nonlinear I-V characteristics of the hypothetical system. We observe nonlinear I-V characteristics with predominantly Ohmic and overlimiting resistance regions. We observe the formation of the ESC region in the overlimiting resistance region. With an increase in applied voltage, the ESC region propagates towards the entire source reservoir and they start to propagate into the nanochannel due to the strong focusing of the normal component of the electric field (see Supplementary Fig. 2). Supplementary Fig. 2b-c shows the snapshots of the hypothetical (without considering water molecules) MD simulations for the applied voltage of 0.5V and 5V, respectively. We do not observe convective vortices near the nanochannel owing to the absence of the water molecules (see Supplementary Movie. 5–6). We infer from these observations that the charge redistribution may be the true mechanism for the overlimiting resistance region and not the strong convective vortices as postulated by earlier experiments and theories.

To demonstrate if the ion selectivity of the nanochannel plays a role on ion concentration polarization, we increased the bulk concentration from 0.1 to 1 M. With an increase in bulk concentration, we observe only the Ohmic region (see Supplementary Fig. 4) because the nanochannel is no longer ion selective as the EDL remains local to the nanochannel surface at 1M concentration^20–23^. Similarly, the recent study shows only the Ohmic behaviour when the surface charge density of the nanochannel becomes zero (as no EDL formation)^34^. Typically, the communication between the nanochannel wall atoms and ions are established through van der Waals (vdW) and electrostatic interactions. To understand the interplay between these interactions on the ion concentration polarization, we reduced the vdW interactions of the nanochannel wall atoms by 10 times. The results revealed that the interaction between the nanochannel wall atoms and ions is purely electrostatic (see Supplementary Fig. 5) and we observe the same I-V characteristics. Thus, we highlight that the ion selectivity of the nanochannel (controlled by surface charge density and the ionic concentration) plays a dominant role in controlling the ion concentration polarization near the nanochannel interconnect devices.

## Anomalous water compression effect

**Figure 4:**
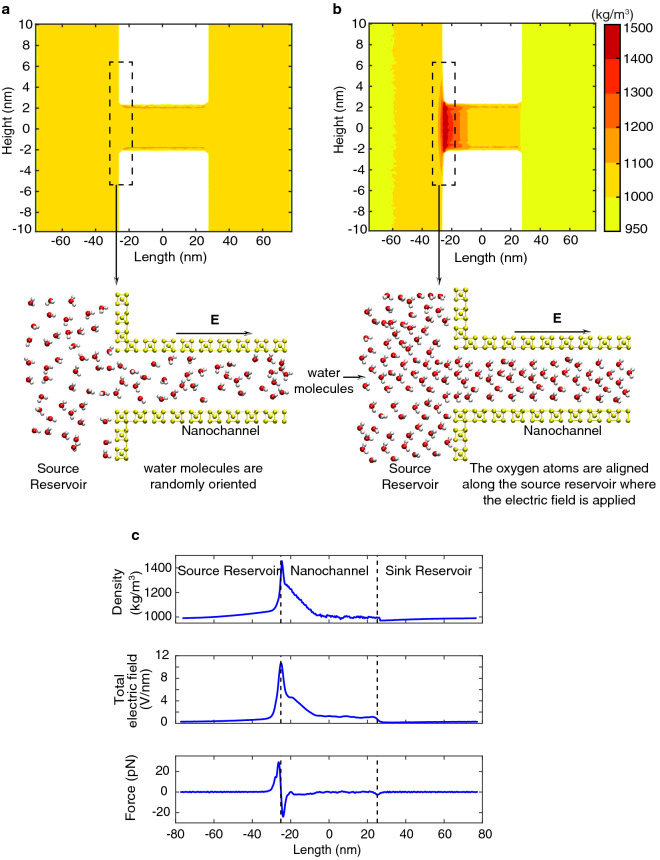
(**a, b**) shows the density distribution of the water molecules corresponding to the applied voltage of 30V and 170 V. Further, it shows the corresponding snapshots of the all-atom MD simulations. For clear visualizations, we showed one layer of water molecules. (**c**) shows a comparison of 1D distribution of water density, total electric field and dielectrophoretic force acting on the water molecules for the applied voltage of 170V. Drawings in (**a**, **b**) were created using VMD software (www.ks.uiuc.edu/Development/Download/download.cgi?PackageName=VMD and version 1.9.4).

Recent MD simulations of DNA transport through graphene nanopores reported local compression of water under a strong electric field^48^. Fig. 4a-b shows the density distribution of the water molecules corresponding to the applied voltage of 30V and 170 V. We observe a uniform density of water throughout the system in the Ohmic region (see Fig. 4a). But in the overlimiting resistance region, we observe an increase in the density of water at the interface of the nanochannel and source reservoir (see Fig. 4b). The formation of the ESC region at the interface of the nanochannel and source reservoir creates a non-uniform electric field along the length of the nanochannel. The non-uniform electric field imposes a non-zero net force on the water molecules. The non-zero net force acting on the water molecule causes a dielectrophoretic force, which is a product of the gradient of the total electric field and the dipole moment of the water molecule,

2 $$\begin{aligned} F_z = d \nabla E^{total}_z \end{aligned}$$

where, d is the dipole moment of the water molecule and *z* shows along z direction. For SPC/E rigid water molecule, the dipole moment is 2.35 D (D - debyes). Fig. 4c shows the 1D density distribution of water, non-uniform electric field distribution and the dielectrophoretic force acting on the water molecules, corresponding to the applied voltage of 170 V (overlimiting resistance region). It is evident that the density of water reaches the maximum value at the same location where the electric field is maximum. The presence of dielectrophoretic force leads to anomalous compression of water molecules near the interface of the nanochannel (see Fig. 4c). Furthermore, the dielectrophoretic force aligns the water molecules along the direction of the applied electric field (The oxygen atoms are facing the source reservoir, whereas the hydrogen atoms are facing the sink reservoir see Fig. 4a–b snapshots from all-atom MD simulations). The anomalous water compression effect may play an important role in trapping DNA molecules and any other biomolecules near the nanochannel^48^.

## Conclusions

In this paper, we have shown that the charge redistribution mechanism near the nanochannel may be the true mechanism for the origin of the overlimiting current. Further, the convective vortices may not necessarily be the true mechanism for overlimiting current as postulated by earlier experiments and theories. Our work may help devise new theories for ion concentration polarization and overlimiting current near the nanochannel interconnect devices.

## Methods

### Molecular dynamics simulation setup

A cubic unit cell of $\gamma$-silicon nitride crystal (${\gamma }$-${Si_3N_4}$) was replicated in three dimensions to construct a substrate of 4.64 x 20.1 x 50.27 nm dimension^9^. A nanochannel of height 5 nm was created by removing the atoms of the substrate with respect to its centre (see Fig. 1c). The removal of the atoms from the substrate led to a non-zero charge of the substrate. To maintain electro-neutrality of the surface atoms, the partial charge of the SI atom was adjusted by 0.1% such a way the total charge of the system is zero. In this work, we assume that the nanochannel has a uniform negative surface charge ($\sigma$) of ${-0.6} \, {C/m^2}$. In order to make the nanochannel with desired negative surface charge, additional charges were added to the surface atoms of the nanochannel along with its partial charge. The partial charges of the atoms other than the surface of the nanochannel are assumed to be zero since we were not interested in the interactions between the atoms of the substrate. The entire system was immersed in a pre-equilibrated KCl electrolyte solution of 0.1 M concentration, and additional potassium (${K^+}$) ions were added to maintain electro-neutrality of the system (see Supplementary Table. 1). The whole system is assumed to be periodic in all three dimensions.

Water molecules and ions were modelled using the SPC/E model and LJ particles, respectively. The non-bonded LJ potentials of the all-atom types were given in Supplementary Table. 2. Further, the bonds between the hydrogen and oxygen atoms in the water molecule were maintained rigidly. The cut-off distance of 1.2 nm with a switching function starts at 1 nm was used to calculate the non-bonded interactions among the atoms. Also, the particle mesh Ewald (PME) algorithm with a grid space of 0.12 nm was employed for the electrostatic interactions. The temperature of the system is controlled to 300 K by Langevin dynamics, and a damping coefficient of $0.1 \, {ps^{-1}}$ was applied only to the water molecules ( see Supplementary Note. 1). Finally, ${Si_3 N_4}$ substrate atoms were fixed in space and excess atoms in the substrate except two layers of surface atoms were removed to reduce the computational cost and memory storage problem.

All the MD simulations were performed using the NAMD 2.12^49^ computational engine with a multi-time step (MTS) algorithm having a 1-2-4 fs time step. VMD^50^ was used for visualizing and movie-making purposes. The trajectories and velocities of the MD simulations were written for every 0.1 ps and were analyzed using custom TCL and C++ scripts.

Initially, the MD simulation system was minimized for 1000 steps using the conjugate gradient method to avoid local contacts between the atoms and to bring the atoms to its local minimum energy state according to their force field. After the minimization, the system was equilibrated for 5 ns in NPT ensemble using Langevin piston barostat by allowing fluctuation only in z-direction in order to maintain uniform density throughout the system, and followed by NVT ensemble for 10 ns to perform equilibrium calculations. Finally, non-equilibrium simulations were performed for 10 ns in the NVT ensemble with a voltage range of 5 V - 200 V using the following relation (Eq.(3)). The uniform electric field was applied perpendicular to the nanochannel (z-direction) to all atoms of the system^9,51,52^.

3 $$\begin{aligned} E^{applied} = \frac{V}{L_z} \end{aligned}$$

where, ${E^{applied}}$ is the uniform electric field applied along z direction, *V* is the applied voltage (volts), and ${L_z}$ is the length of the system along z axis.

Our MD procedure and the post processing codes are validated with the literature and given in Supplementary Fig. 6.

For without water (hypothetical) MD simulations, the dielectric constant is set to the value of 79. The thermostat is coupled to the ions with a dampening coefficient of $20 \, ps^{-1}$ to maintain the temperature of 300 K. Remaining protocols are kept the same as the actual all-atom MD simulations.

### Computation of concentration and velocity profiles

The concentration and velocity profiles are calculated by using binning method and particle tracking method, respectively^38,39^.

### Computation of total electric potential

When a uniform electric field (Eq. 3) is applied to all atoms of the MD simulation, charged atoms will be redistributed inside the system to nullify the external electric field. Therefore, the total electric potential is a combination of the reaction potential (${\varphi ^{reaction}}$ - due to the position of the charged atoms) and applied potential (${\varphi ^{applied}}$ - linear along the length of the system). Total electric potential along the length is given by^53^,

4 $$\begin{aligned} \varphi ^{total} = \varphi ^{reaction} + \varphi ^{applied} \end{aligned}$$

The reaction potential is calculated by using VMD PMEpot plugin^54^. The 3D reaction potential map from the plugin is averaged in one and two dimensional profiles. The applied potential is given by,

5 $$\begin{aligned} \varphi ^{applied} = (z - z_{min}) E^{applied} \end{aligned}$$

where, ${z_{min}}$ is the minimum position in z direction (coordinate of the left extreme point).

### Computation of total electric field

Total electric field is the gradient of the total electric potential and is given below,

6 $$\begin{aligned} E^{total} = - \nabla \varphi ^{total} \end{aligned}$$

## Supplementary information

Supplementary Information

Supplementary Movie 1

Supplementary Movie 2

Supplementary Movie 3

Supplementary Movie 4

Supplementary Movie 5

Supplementary Movie 6
