# Supplementary material for: Overlimiting current near a nanochannel a new insight using molecular dynamics simulations
Source: Sci Rep. 2021 Jul 26;11:15216. doi: 10.1038/s41598-021-94477-x (PMC8313724; doi:10.1038/s41598-021-94477-x)
Supplement: Supplementary file 1 — Supplementary Information [file 41598_2021_94477_MOESM1_ESM.pdf]

Supplementary Information : Overlimiting current near a nanochannel a new insight using molecular dynamics simulations

D. Manikandan[1] and Vishal V.R. Nandigana[1,*]

[1] *Fluid Systems Laboratory, Department of Mechanical Engineering,*

Indian Institute of Technology Madras, Chennai 600036, India

[*]All correspondence and request for materials should be addressed to *nandiga@iitm.ac.in*

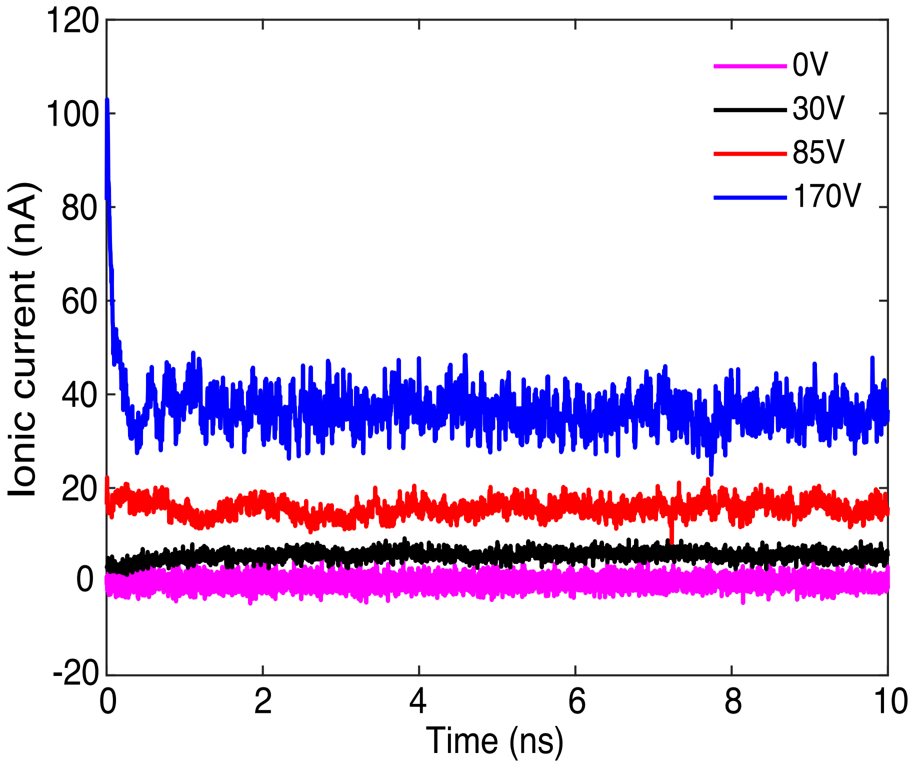

Supplementary Figure. 1. Variation of current with time. Initially, current decays with time and after 1-2 ns current fluctuates around the steady-state value. The zero voltage case refers to the NVT equilibrium simulation.

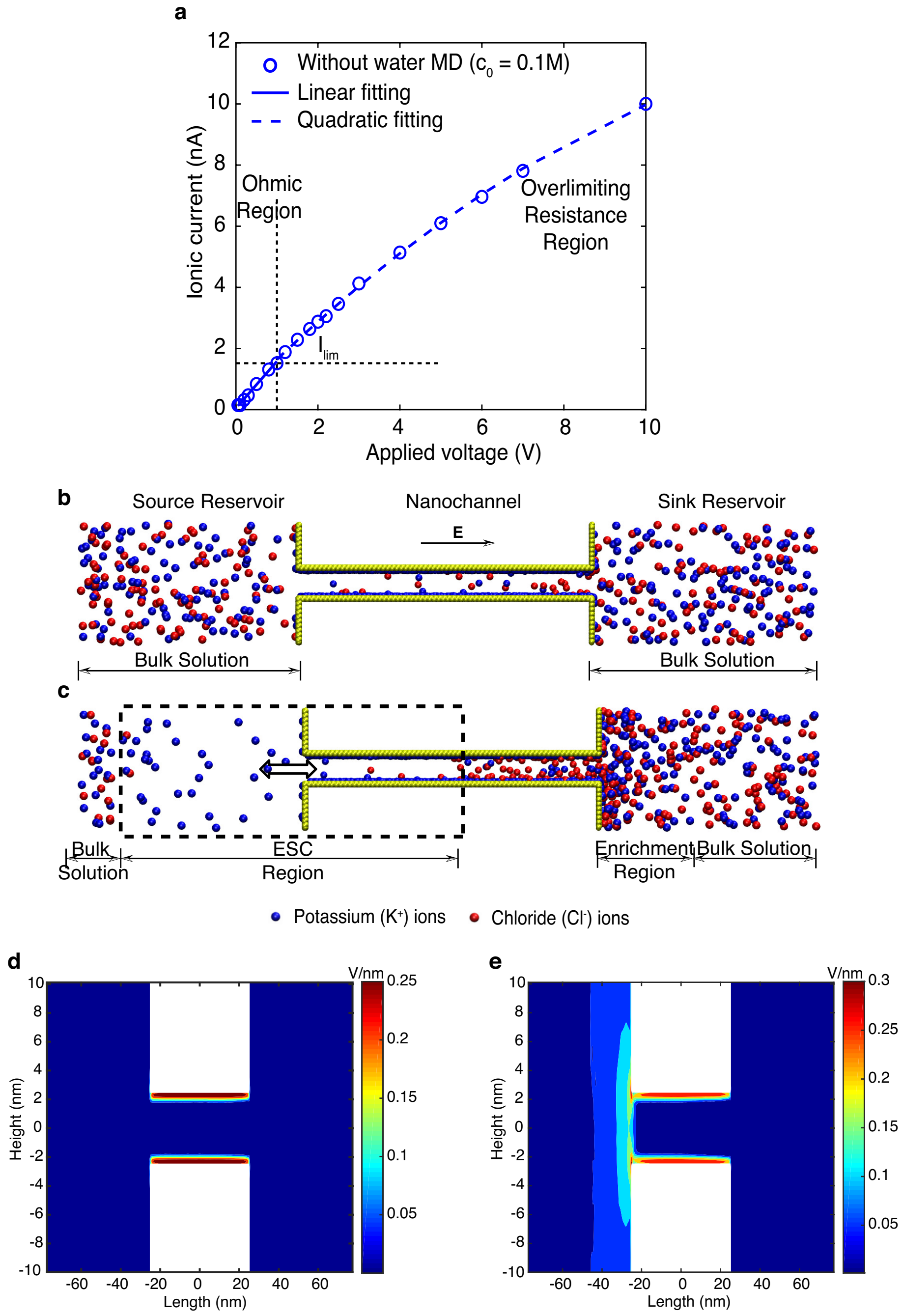

Supplementary Figure. 2. **a.** the nonlinear I-V characteristics of the without water (hypothetical) MD simulations. **b-c.** shows the snapshots of the without water (hypothetical) MD simulations corresponding to the applied voltages of 0.5V and 5V, respectively. **d-e.** 2D total electric field distribution for the same voltages. We see charge redistribution inside the nanochannel owing to the focusing of the electric field even in the absence of the strong convective vortices. Drawings in Supplementary Figure.2b-c were created using VMD software (www.ks.uiuc.edu/Development/Download/download.cgi?PackageName=VMD and version 1.9.4).

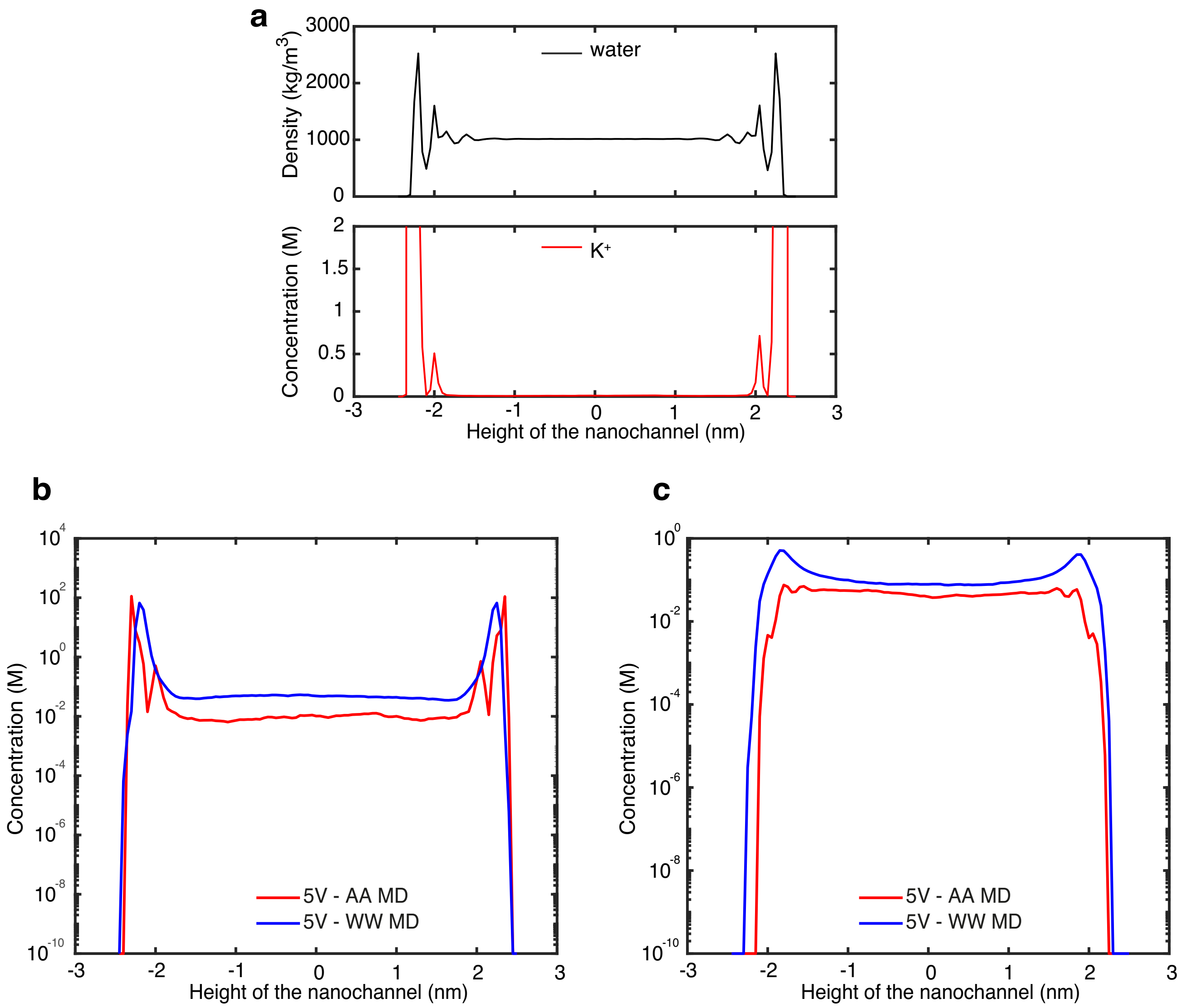

AA MD - All atom molecular dynamics
WW MD - Without water (hypothetical) molecular dynamics

Supplementary Figure. 3. **a.** shows the comparison of the density of water and cation concentration inside the nanochannel along the height of the channel. We see an increase in density of the water molecules near the wall of the nanochannel then it decreases and again increases and reaches the uniform density. We observe a similar behaviour near the wall of the nanochannel for the potassium (K^+) ions owing to the discrete nature of the water molecules and ions. **b-c.** shows the comparison of the potassium (K^+) and chloride (Cl^-) ions between the all-atom and without water (hypothetical) MD simulations, corresponding to the applied voltage of 5 V, respectively.

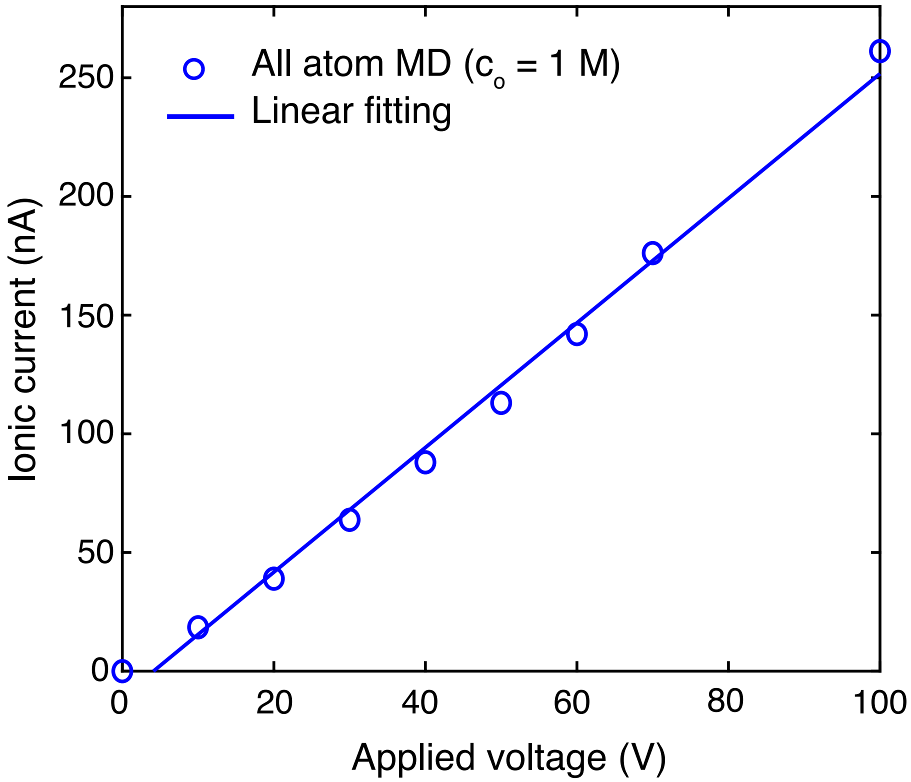

Supplementary Figure. 4. shows I-V characteristics of the system for 1M concentration. With an increase in concentration, we observe only the Ohmic region because the nanochannel is no longer ion selective as the EDL remains local to the nanochannel surface at 1M concentration.

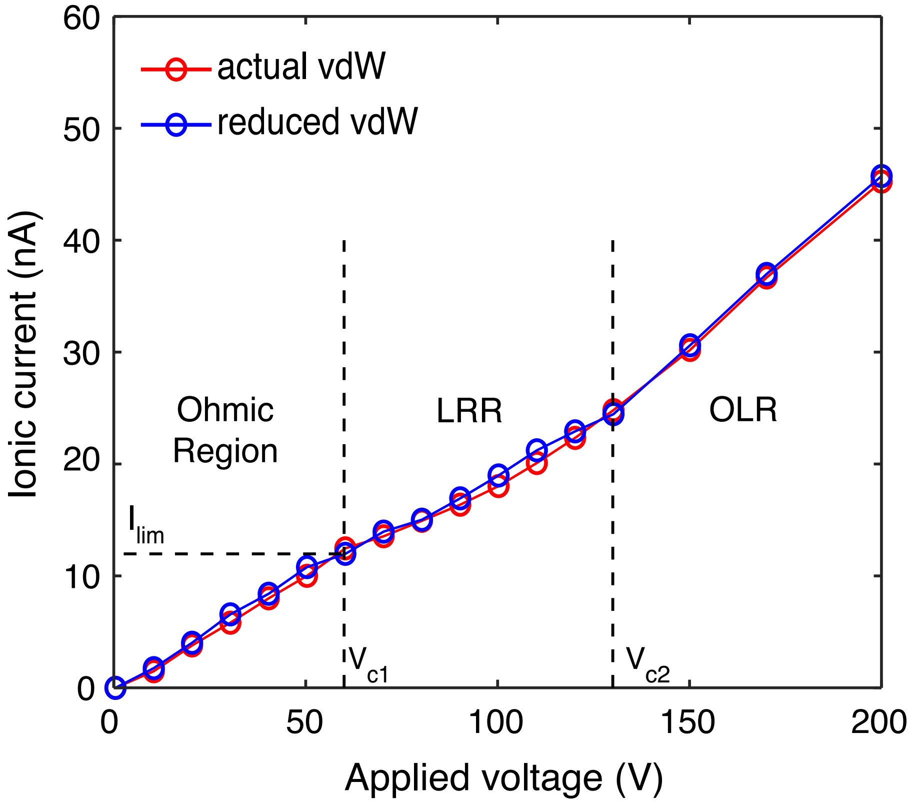

Supplementary Figure. 5. shows I-V characteristics for the actual and reduced vdW interaction strength of Si and N atoms of the nanochannel. We have observed the same I-V characteristics when reducing the vdW interaction strength for Si and N atoms. The results revealed that the interaction between the nanochannel wall atoms and the ions is purely electrostatic due to the surface charge density of the nanochannel.

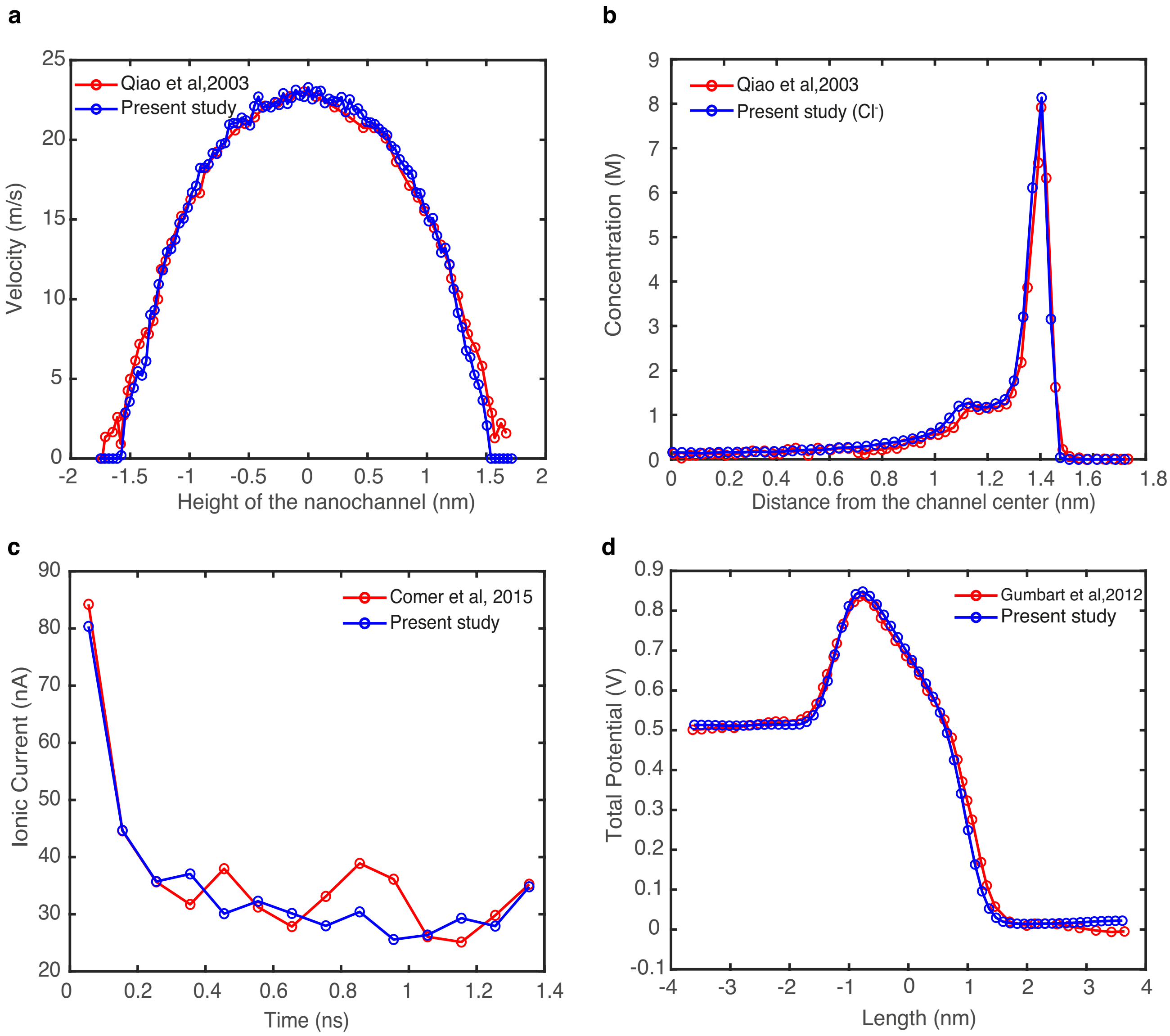

Supplementary Figure. 6. The proposed MD model and the post-processing codes are all validated with the literature. The results are found to be in close agreement with the literature results[1–3].

Supplementary Table. 1. Details of atoms in the system

Description of atoms	Number of atoms (#)
Silicon nitride	21702
water (including H and O atoms)	1103649
Potassium ions (K^{+})	2140
Chloride ions (Cl^{-})	662
Total number of atoms	1128423

Supplementary Table. 2. Force field parameters for LJ potentials in CHARMM format

Types of atoms	ϵ (kcal/mol)	$R_{min}/2$ ($\mathring{A}$)
Si [4,5]	-0.31000	2.13500
N [4,5]	-0.19000	1.99750
OH [6]	-0.155290	1.76282
H [6]	-0.00000	0.00010
K [7]	-0.42970	1.59300
Cl [7]	-0.01278	2.71100

* Interactions between the atom types are calculated by Lorentz-Berthelot rules

Supplementary Note 1 : Effect of the thermostat coupling

The controlling of temperature is a crucial part of the molecular dynamics simulations, and it plays an important role in controlling the dynamics of the system within a short time period. Here, the controlling of temperature can be done by coupling the atoms of the system to the large heat bath or reservoir, such that instantaneous kinetic energy of the coupled system corresponds to a target temperature[8]. Mostly, this kind of coupling is done by modifying Newton's equation of motion or rescaling the atom velocities. In our case, we employed the Langevin dynamics method to control the temperature of the system. Langevin dynamics method adds the additional frictional force to Newton's equation of motion rather than rescaling the velocities of the atoms. The modified equation of motion is,

$$m_i \frac{d^2 r_i}{dt^2} = -m_i \gamma \frac{dr_i}{dt} + F_i(r_i) \tag{S1}$$

Where r is the position of the atom, m is the mass of the atom, i = 1 to N, N is the total number of atoms, F is the force acting on the atoms due to electrostatic and nonbonded interactions and γ is the damping coefficient ($\gamma = 1/\tau$,τ is the relaxation time). The large value of the damping coefficient indicates a strong coupling of the thermostat, whereas the small value shows a weak coupling of the thermostat. Recently, it has shown that the dynamics and the properties of the system are dampened by the strong coupling of the thermostat than the weak coupling[8]. Also, it has observed that the actual properties of the system are maintained through the weak coupling of the thermostat[8]. Further, to understand the effect of the weak and strong coupling of the thermostat on the concentration distribution and ionic current, we performed additional simulations for damping coefficient values of 0.1, 1, 20 ps^{-1} corresponding to the applied voltage of 150 V.

Supplementary Fig. 7a,b shows the concentration distribution of ions along the length of the system, corresponding to the damping coefficient of 0.1 ps^{-1} (weak coupling) and 20 ps^{-1} (strong coupling) respectively. We observe the formation of the ESC region at the interface of the nanochannel and source reservoir, similarly, the enrichment region at the interface of the nanochannel and sink reservoir. For the weak coupling of the thermostat, the ESC region expands inside the source reservoir and occupies almost the entire source reservoir whereas it remains local to the interface of the nanochannel and source reservoir in case of the strong coupling. Similar behaviour is seen for the enrichment region in both cases.

Further, Supplementary Fig. 7e shows the comparison of the ionic current values for these three damping coefficients. We see suppression of the ionic current for the strong coupling of the thermostat as compared to the weak coupling. We almost observe 68% decrease in the ionic current for the strong coupling than the weak coupling of the thermostat. This decrease in the ionic current is seen because of the restriction in the expansion of the ESC region. Also, Supplementary Fig. 7c,d shows the snapshot of the all-atom MD simulations corresponding to the weak and strong coupling of the thermostat, respectively. It shows a clear picture of the restriction of the propagation of the ESC region.

From these discussions, we infer that the strong coupling of the thermostat dampens the dynamics of the system and also changes the behaviour of the system. In order to simulate the reasonable dynamics and behaviour of the system, the weak coupling of the thermostat is used throughout this paper.

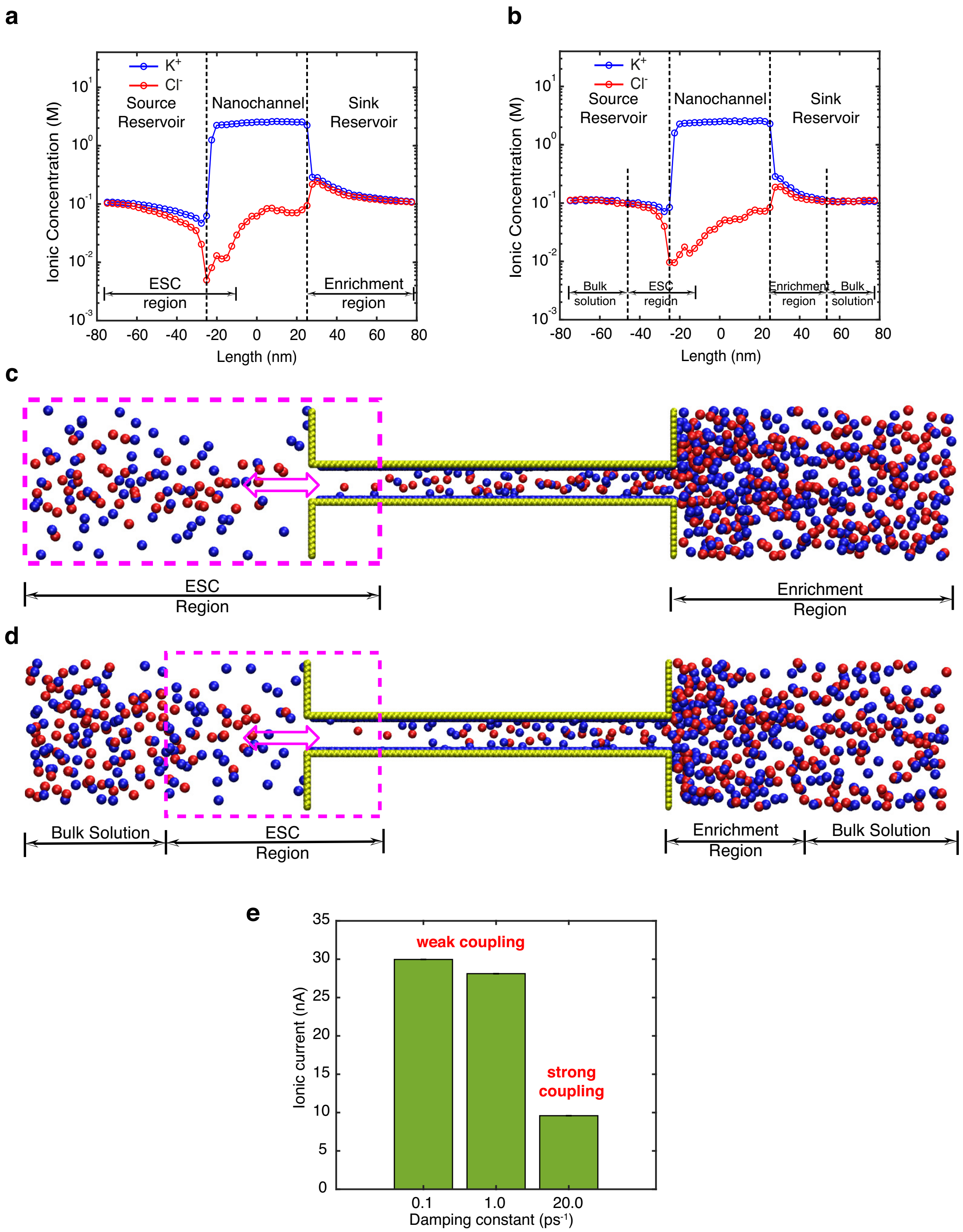

Supplementary Figure. 7. a-b. shows the concentration distribution of the ions along the length of the system for the applied voltage of 150 V, corresponding to the damping coefficient of 0.1 ps^{-1} and 20 ps^{-1}, respectively. **c-d.** shows the snapshot of the all-atom MD simulations for 0.1 ps^{-1} and 20 ps^{-1}, respectively. **e.** shows the comparison of the ionic current for all three damping coefficients. Drawings in Supplementary Figure.7c-d were created using VMD software (www.ks.uiuc.edu/Development/Download/download.cgi?PackageName=VMD and version 1.9.4).

Description of Additional Supplementary Files

Supplementary Movie. 1

Last 2 ns of all-atom MD simulation of the Ohmic region corresponding to the applied voltage of 30 V. For clear visualization, we removed water molecules and showed only ions.

Supplementary Movie. 2

Last 2 ns of all-atom MD simulation of limiting resistance region (LRR) corresponding to the applied voltage of 85 V. For clear visualization, we removed water molecules and showed only ions.

Supplementary Movie. 3

Last 2 ns of all-atom MD simulation of overlimiting resistance region (OLR) corresponding to the applied voltage of 170 V. For clear visualization, we removed water molecules and showed only ions.

Supplementary Movie. 4

The formation of the counter-rotating vortices inside the source reservoir for overlimiting resistance region (OLR) corresponding to the applied voltage of 170 V. For clear visualization, we changed the colour of certain water molecules to see the movement of water molecules.

Supplementary Movie. 5

Last 2 ns of without water (hypothetical) MD simulation of the Ohmic region corresponding to the applied voltage of 0.5 V.

Supplementary Movie. 6

Last 2 ns of without water (hypothetical) MD simulation of overlimiting resistance region (OLR) corresponding to the applied voltage of 5 V.

1. Qiao, R. & Aluru, N. R. Ion concentrations and velocity profiles in nanochannel electroosmotic flows. *The Journal of Chemical Physics* **118**, 4692–4701 (2003).
2. Comer, J., Wells, D. B. & Aksimentiev, A. *Modeling nanopores for sequencing DNA*, vol. 749, 317–358 (2011).
3. Gumbart, J., Khalili-Araghi, F., Sotomayor, M. & Roux, B. Constant electric field simulations of the membrane potential illustrated with simple systems. *Biochimica et Biophysica Acta (BBA) - Biomembranes* **18**, 294 – 302 (2012).
4. Wendel, J. A. & Goddard, W. A. The hessian biased force field for silicon nitride ceramics: Predictions of thermodynamic and mechanical properties for α - and β - Si_3N_4. *The Journal of Chemical Physics* **97**, 5048–5062 (1992).
5. Aksimentiev, A., Heng, J. B., Timp, G. & Schulten, K. Microscopic kinetics of dna translocation through synthetic nanopores. *Biophys J* **87**, 2086–97 (2004).
6. Berendsen, H. J. C., Grigera, J. R. & Straatsma, T. P. The missing term in effective pair potentials. *The Journal of Physical Chemistry* **91**, 6269–6271 (1987).
7. Joung, I. S. & Cheatham, T. E. Determination of alkali and halide monovalent ion parameters for use in explicitly solvated biomolecular simulations. *The Journal of Physical Chemistry B* **112**, 9020–9041 (2008).
8. Basconi, J. E. & Shirts, M. R. Effects of temperature control algorithms on transport properties and kinetics in molecular dynamics simulations. *Journal of Chemical Theory and Computation* **9**, 2887–2899 (2013).
